# 2212 A systematic overview of cost-utility analyses in dermatology

**DOI:** 10.1017/cts.2018.42

**Published:** 2018-11-21

**Authors:** David G. Li, Adam Faletsky, Peter Neumann, Joshua Cohen

**Affiliations:** 1 School of Medicine, Tufts University; 2 Tufts University

## Abstract

OBJECTIVES/SPECIFIC AIMS: Costs associated with the treatment of skin diseases accounted for greater than 4% of total US healthcare spending in 2013, an increase of $46 billion (170%) since 2004. Considering the increase in novel treatments and spending, cost-utility analyses (CUAs) may provide a better understanding of costs in dermatology. In this study, we conduct a systematic overview of study quality among CUAs related to dermatology. METHODS/STUDY POPULATION: We queried studies from the Tufts Medical Center Cost-Effectiveness Analysis Registry (www.cearegistry.org), a database supplying information on all peer-reviewed cost-effectiveness analysis through 2014. Database methodology was previously discussed here. We queried studies using keywords from the 24 major skin disease categories (e.g., diseases relating to actinic damage were searched by using “actinic,” “actinic keratosis”). We collected data on study design, reporting methods, and analyzed relevant data stratified by 2 time-periods (1976–2008 and 2009–2014) chosen to encompass a comparable number of studies. RESULTS/ANTICIPATED RESULTS: In total, 42 and 50 studies corresponding to the 2 time-periods were retrieved (representing 14/24 disease categories). Based on the recommended data reporting guidelines for CUAs, study quality remained largely unchanged across the 2 phases. Across the 2 time-periods, a societal perspective was used in 19% and 12% of studies, costs and (quality adjusted life-years) QALYs were discounted in 67% and 72% of studies, a correct (incremental cost-effectiveness ratio) ICER was reported in 67% and 72% of studies, and a sensitivity analysis was included in 88% and 84% of studies, respectively. DISCUSSION/SIGNIFICANCE OF IMPACT: Our findings suggest the quality of dermatology-related CUAs, as evaluated by recommended data reporting guidelines, to be generally stable during the analyzed time-periods. However, the quality of our results may be limited by the small number of CUAs within dermatology (10/24 disease categories did not have CUAs across any time-period). Moving forward, we encourage researchers within dermatology to pursue additional investigation towards cost-effective practices while adhering closely to recommended quality reporting guidelines for CUAs.

